# The Role of Epithelial to Mesenchymal Transition in Human Amniotic Membrane Rupture

**DOI:** 10.1210/jc.2016-3150

**Published:** 2016-12-19

**Authors:** Carla Janzen, Suvajit Sen, Margarida Y. Y. Lei, Marina Gagliardi de Assumpcao, John Challis, Gautam Chaudhuri

**Affiliations:** 1 Department of Obstetrics and Gynecology, David Geffen School of Medicine, University of California 90095, Los Angeles, Los Angeles, California; 2 Faculty of Medical Sciences of Santa Casa de Sao Paulo, Sao Paulo, 01221-020, Brazil; 3 University of Western Australia and Curtin University, Crawley, Western Australia 6009, Australia

## Abstract

**Context:**

Biochemical weakening of the amnion is a major factor preceding preterm premature rupture of membranes (PPROMs), leading to preterm birth. Activation of matrix metalloproteinases (MMPs) is known to play a key role in collagen degradation of the amnion; however, epithelial to mesenchymal transition (EMT) that is also induced by MMP activation has not been investigated as a mechanism for amnion weakening.

**Objective:**

To measure amniotic EMT associated with vaginal delivery (VD) compared with unlabored cesarean sections (CSs), and to assess changes in amniotic mechanical strength with pharmacologic inhibitors and inducers of EMT, thus testing the hypothesis that EMT is a key biochemical event that promotes amniotic rupture.

**Findings:**

(1) Amnions taken from VD contained a significantly increased number of mesenchymal cells relative to epithelial cells compared with unlabored CS by fluorescence-activated cell sorting analysis (60% vs 10%); (2) tumor necrosis factor (TNF)–*α* stimulation of amniotic epithelial cells increased expression of the mesenchymal marker vimentin after 2 days; (3) EMT inhibitor, etodolac, significantly increased the time and mechanical pressure required to rupture the amnion; and (4) TNF-*α* and another pharmacologic EMT inducer, ethacridine, decreased the time and mechanical pressure required for amnion rupture, further confirming that the mesenchymal phenotype significantly weakens the amnion.

**Conclusions:**

This work demonstrated amniotic cell EMT was associated with labor and EMT decreased the tensile strength of the amnion. These findings suggest a role for EMT in the pathophysiology of PPROM and may provide a basis for development of therapies to prevent preterm labor.

## Teaser

We evaluated EMT in the human amnion and found that labor and PPROM are associated with increased amniotic EMT compared with unlabored cesarean delivery.

The precise biochemical mechanism by which preterm or term rupture of amniotic membrane occurs during pregnancy is not yet known; however, various potential mechanisms have been proposed. The rupture mechanism was long thought to be a consequence of uterine contractions. However, observation of an amniotic zone of altered morphology in the region that overlies the cervix that contains increased apoptosis, modifications of metalloproteinase, and proteoglycan activity, in association with membrane weakness, suggests that there may be programming of the rupture of the amnion before parturition (1). The participation of a mechanical factor as the only cause of rupture of fetal membranes during normal labor or premature rupture has therefore been criticized, and the involvement of an enzymatic mechanism has been proposed. It has been demonstrated that term amniotic fluids are capable of inducing the synthesis of collagenase and other proteases in fibroblasts, as revealed by selective increases in collagenase activity and in immune-reactive collagenase. Nonterm amniotic fluids however failed to do the same. This phenomenon was therefore proposed as a model for studying the collagen degradation of fetal membranes during term gestation (2). Separate cell culture from different layers of fetal membranes and culture of purified placental trophoblast cells showed that placental syncytiotrophoblast and amnion epithelial cells exclusively produced matrix metalloproteinase-9 (MMP-9); chorion trophoblast cells secreted both MMP-2 and MMP-9, but amnion mesenchymal cells produced only MMP-2. It was therefore concluded that MMP-2 and MMP-9 exhibited cell-specific expression in the human placenta (3). On these bases, it was further suggested that an increase in MMP-9 expression may contribute to the degradation of the extracellular matrix in the fetal membrane and placenta, thereby facilitating fetal membrane rupture and placental detachment from the maternal uterus at labor and both preterm and term (3). Subsequently, it was demonstrated that human placenta and fetal membranes expressed an extracellular MMP inducer EMMPRIN, with the potential to stimulate MMP production, thereby facilitating fetal membrane rupture and leading to detachment of the placenta and fetal membranes from the maternal uterus at the time of parturition (4).

Detachment of epithelial cells from the surrounding tissue is a common event between cell invasion and metastasis in cancer, and a similar phenomenon occurs during the rupture of the amniotic membrane during fetal delivery (5–8). The key biochemical event that is known to cause epithelial detachment in cancer is epithelial to mesenchymal transition (EMT) (5, 6). Thus, we hypothesize that a similar phenomenon could account for the rupture of amniotic membrane. The main features accompanying this mechanism are the loss of epithelial characteristics of cells and the acquisition of mesenchymal markers, such as fibronectin, vimentin, and *N*-cadherin (5, 6). Interestingly, the major cytokines and signaling mediators that promote EMT in cancer, including tumor necrosis factor (TNF)–*α*, interleukin-6, interleukin-8, prostaglandins, and MMP-9, are also found to be biologically active and in substantial concentrations in the fetoplacental unit (9–11). Moreover, several phenotypic manifestations associated with mesenchymal transition, such as disorganization of the cytoskeleton, disruption of intercellular adhesions, and degradation of the extracellular matrix, as described in cancer, are also the major biological ramifications leading to the rupture of amniotic membranes (7, 8). Despite these striking similarities, the possibility of EMT in the amniotic epithelial cells, being causal and at least in part responsible for the rupture of the amniotic membrane rupture, was unclear.

In view of the evidence that MMP may play an important role in the rupture of amniotic membrane, and the emerging fact that MMPs can also stimulate the processes associated with EMT (3, 4, 12), we evaluated the role of EMT as an important mechanism involved in the rupture of the amniotic membrane. In this study we tested the hypothesis that EMT is a key biochemical event that promotes amniotic membrane rupture.

## Materials and Methods

### Amnion collection and processing

Informed consent under the approval of the University of California, Los Angeles, Institutional Review Board was obtained from women with normal pregnancy that delivered by scheduled unlabored cesarean section (CS) or vaginal delivery (VD) and from those affected by preterm premature rupture of membrane (PPROM). Term placentas with attached fetal membranes were collected immediately after delivery. All manipulations were carried out under sterile conditions. The amnion was peeled from the chorion and washed in Dulbecco’s phosphate-buffered saline, pH 7.5 (Thermo Fisher Scientific, Rockford, IL), before subjected to experimental conditions.

### Isolation of amniotic epithelial cells

Amniotic epithelial cells were isolated from freshly obtained amnion as described previously (13, 14). Briefly, the amnion was divided into 2 parts of equal weight. The first amnion part was cut into fine pieces and added to 50 mL of 1 mg/mL collagenase. It was then shaken at 37°C for 2 hours. The amnion/collagenase mixture was then filtered through a 100-µm nylon mesh and centrifuged into a pellet at 2500*g* for 10 minutes. The pellet was suspended in 3 mL of Dulbecco’s modified Eagle medium (DMEM) and then layered on a discontinuous Percoll gradient. The gradient was then centrifuged at 800*g* for 20 minutes. A band of cells was collected at the 20% Percoll level. The cells were then suspended in DMEM with fetal bovine serum and a mixture of 1000 U/mL penicillin, 0.1 mg/mL streptomycin, and 0.23 µg/mL amphotericin B.

### Isolation of mesenchymal cells

The second amnion part was placed into a solution of 0.25% trypsin in DMEM and shaken at 37°C for 20 minutes. The supernatant from this first incubation was discarded. The amnion was then incubated 2 more times in 0.25% trypsin at 37°C for 30 minutes each time. The supernatant from those 2 incubations was collected and centrifuged into a pellet at 2500*g* for 10 minutes. The pellets from both amnion parts were suspended in 3 mL of DMEM and then layered on a discontinuous Percoll gradient. The gradient was then centrifuged at 800*g* for 20 minutes. A band of cells was collected at the 20% Percoll level. The cells were then suspended in DMEM with fetal bovine serum and a mixture of 1000 U/mL penicillin, 0.1 mg/mL streptomycin, and 0.23 µg/mL amphotericin B. Cell viability was measured by a Vi-Cell Viability Analyzer (BD Biosciences, Franklin Lakes, NJ). Both sets of cells were >90% viable.

### Flow cytometry (fluorescence-activated cell sorting)

Freshly isolated epithelial and mesenchymal cells were combined for fluorescence-activated cell sorting (FACS) analysis. The freshly isolated combined epithelial and mesenchymal cells were stained with fluorescein isothiocyanate antihuman E-cadherin (BioLegend, San Diego, CA) and the phycoerythrin (PE) antihuman vimentin (BD Pharmingen, Franklin Lakes, NJ) antibodies. The isotype control antibodies were used at the same concentrations according to the manufacturer’s instructions. Cells were washed with phosphate-buffered saline before analysis using a FACSCalibur (BD Biosciences).

### Cell culture

The freshly isolated primary cells were plated and maintained in culture at 37°C with a water-saturated atmosphere and 5% CO_2_ (14) until 80% confluent. Epithelial cells were then treated with 10 ng/mL TNF-*α* (Peprotech, Rocky Hill, NJ), wherever applicable, over time as indicated.

### Immunoblot blot analysis

Amnion and epithelial cells were lysed in radioimmunoprecipitation assay buffer with a protease and phosphatase inhibitor cocktail (Thermo Fisher Scientific/Pierce) on ice and centrifuged at 4°C. Supernatants were assayed for protein content using a BCA Protein Assay Kit (Thermo Fisher Scientific). Equal concentrations of samples, 5 μg of tissue lysate protein, and 10 μg of cell lysate protein were loaded onto 10% TGX gels (Bio-Rad, Hercules, CA) and subjected to gel electrophoresis. The contents of the gels were transferred onto polyvinylidene difluoride (PVDF) membranes using Trans-Blot® Turbo™ Transfer System (Bio-Rad). After blocking in 5% bovine serum albumin with 1× phosphate-buffered saline with Tween-20, membranes were incubated with primary antibodies, E-cadherin (Cell Signaling, Danvers, MA) at 1:3000 dilution, vimentin (Cell Signaling) at 1:3000 dilution, *N*-cadherin (Abcam, Cambridge, MA) at 1:3000 dilution, fibronectin (Abcam) at 1:50,000 dilution, and TGF-*β* (Abcam) at 1:3000 dilution, and secondary antibody goat anti-rabbit IgG-horseradish peroxidase at 1:10,000 dilution. Glyceraldehyde 3-phosphate dehydrogenase (Santa Cruz Biotechnology, Dallas, TX) was used as a loading control at 1:10,000 dilution, and secondary antibody goat anti-mouse IgG-horseradish peroxidase was used at 1:10,000 dilution. Immunoreactive signals were analyzed using Pierce ECL Plus (Thermo Fisher Scientific) on a Typhoon Scanner 9410 (GE Healthcare Life Sciences, Pittsburgh, PA) through ImageQuant 5.2 software (GE Healthcare Life Sciences). The protein bands were quantified by densitometry.

### *In vitro* pressure chamber

Then 3- ×3-cm sections of amnion from CS-term pregnancies were prepared from the region measuring 5 cm away from the edge of the placental disk and were incubated in media (no drug control), 10 ng/mL TNF-*α* (Peprotech), 0.5% ethacridine (Abcam), 10^−5^ M Etodolac (Sigma Aldrich, St. Louis, MO), 10 μM celecoxib (Cayman, Ann Arbor, MI), and 10 ng/mL TNF-*α* with 10^−5^ M etodolac at 37°C overnight. The treated amnion was placed to cover the end of a pressurized tube 1 cm in diameter and placed within a DMEM bath. The tube was connected to a syringe pump, which produced pressurized flow of infused DMEM. Membrane rupture was signaled by blue dye from the DMEM entering the DMEM bath. Pressure and time of rupture was recorded. Western blot was performed on all ruptured membranes.

### Data analysis

Statistical analysis was performed using StatView 5.0 software (SAS Institute, Cary, NC). Student *t* test was used to compare 2 groups. When comparing >2 groups, analysis of variance with Fisher protected least significant difference test was used. All data are shown as mean ± standard error of the mean, and *P* < 0.05 was considered statistically significant.

## Results

### Amnion from normal VDs exhibited increased proportion of mesenchymal cells when compared with those from CSs

To test our hypothesis that EMT of amniotic epithelial cells is a key biochemical event that is associated with amniotic membrane rupture, we first tested whether amniotic membranes obtained from normal-term VDs were composed of epithelial cells that have undergone significantly increased mesenchymal transitions, compared with those obtained from unlabored CSs. Elective CS bypasses the normal biochemical transitions associated with the normal-term labor and hence were expected to be composed of epithelial cells with negligible mesenchymal phenotypes. We compared the proportion of epithelial and mesenchymal cells in the amniotic membranes derived from CS with those from VD. To do this, we monitored the relative expressions of E-cadherin (typical marker for epithelial cells) and vimentin (typical marker for mesenchymal cells) to distinguish between epithelial and mesenchymal cells, respectively.

**Figure 1.**
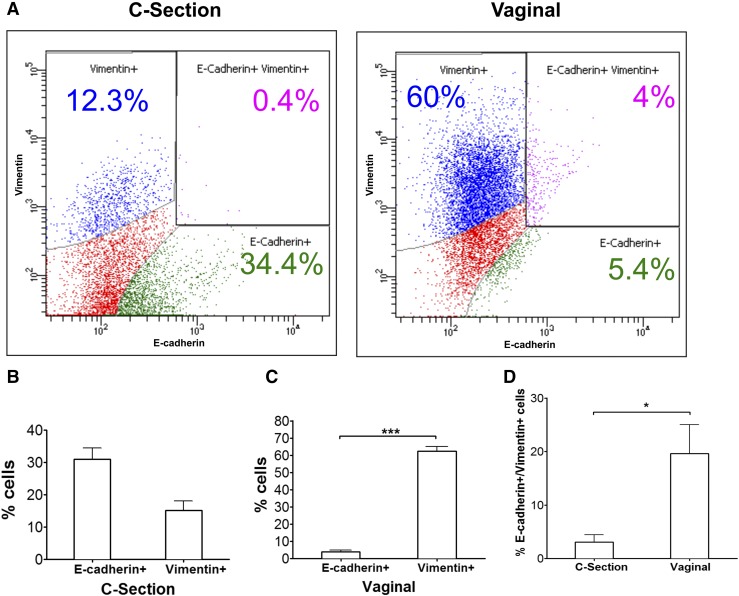
(A) FACS of cell populations obtained from the amniotic membrane taken from CS (left) and VD (right). E-cadherin–fluorescein isothiocyanate and vimentin-phycoerythrin were used as markers for epithelial and mesenchymal cells, respectively, with vimentin+ (blue), E-cadherin+ (green), vimentin+/E-cadherin+ (purple), and unstained (red). (B) The FACS cell populations of E-cadherin+ cells and vimentin+ cells in CS and (C) the amnion from VD (n = 3) are shown. (D) FACS cell populations of double positive E-cadherin+/vimentin+ cells in CS and VD are shown, (n = 3). ***P < 0.0001; *P < 0.05.

Figure 1(A) demonstrates that by FACS, the amnion taken after VD [Fig. 1(A), right upper] exhibited approximately ~60% ± 5% vimentin staining cells (blue), representing mesenchymal cells, compared with only ~12.3% ± 5% taken from CS [Fig. 1(A), left upper]. This figure also demonstrates that VD contained only ~5.4% ± 0.5% E-Cadherin positive/epithelial cells (green) compared with ~34.4% ± 5% epithelial cells in the amniotic membranes from unlabored CS. Relative populations of cells exhibiting E-cadherin, vimentin, and both E-cadherin and vimentin are depicted in green, blue, and purple, respectively [Fig. 1(A)]. Red cells represent unstained cells, presumably endothelial or infiltrating plasma cells. We have not included these negatively staining cells in subsequent analysis, focusing instead on positively staining cells, because E-cadherin and vimentin expression levels have been well established as markers in the study of EMT. Figure 1(B) and 1(C) shows percentages from FACS analysis in graph form. Figure 1(D) shows that there was a significantly increased (>10-fold) population of cells that exhibited both epithelial and mesenchymal markers (purple) derived from VD when compared with those from CS (n = 3; *P* < 0.05).

### Amnion from VD exhibited increased expression of mesenchymal markers when compared with CS

**Figure 2.**
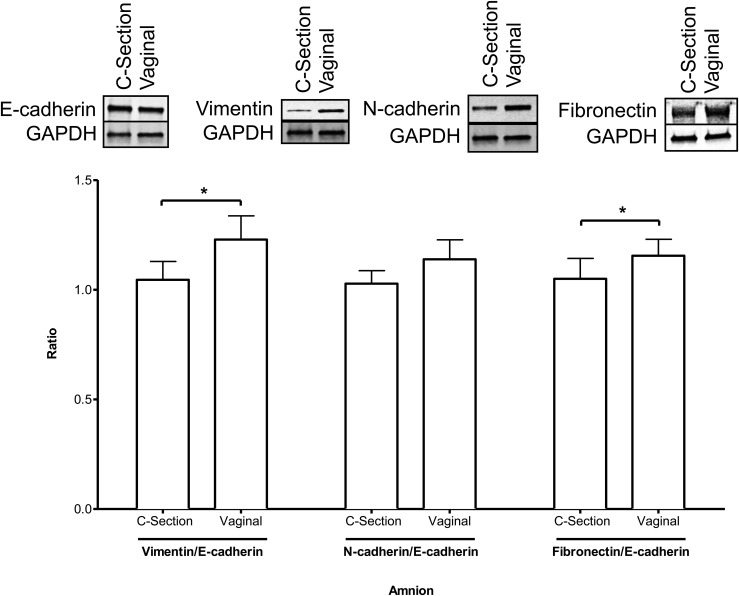
The whole amnion was collected from CS (n = 9) and VD (n = 9). Immunoblot analysis was performed using E-cadherin as a marker for epithelial cells and vimentin, *N*-cadherin, and fibronectin as markers for mesenchymal cells. GAPDH was used as a loading control. *P < 0.05.

Immunoblot analysis to compare epithelial and mesenchymal markers in intact amniotic membranes from VD and CS demonstrated that the epithelial marker E-cadherin was unchanged in VD when compared with CS (Fig. 2, top left). However, there was a substantial increase in mesenchymal markers vimentin and fibronectin in membranes from VD compared with CS (Fig. 2, top). The individual ratios of vimentin, *N*-cadherin, and fibronectin vs E-cadherin, calculated from relative band intensities, were all >1 in VD when compared with CS (n = 9, *P* < 0.05) (Fig. 2).

### Cell culture of amniotic epithelial cells increases vimentin expression over time

**Figure 3.**
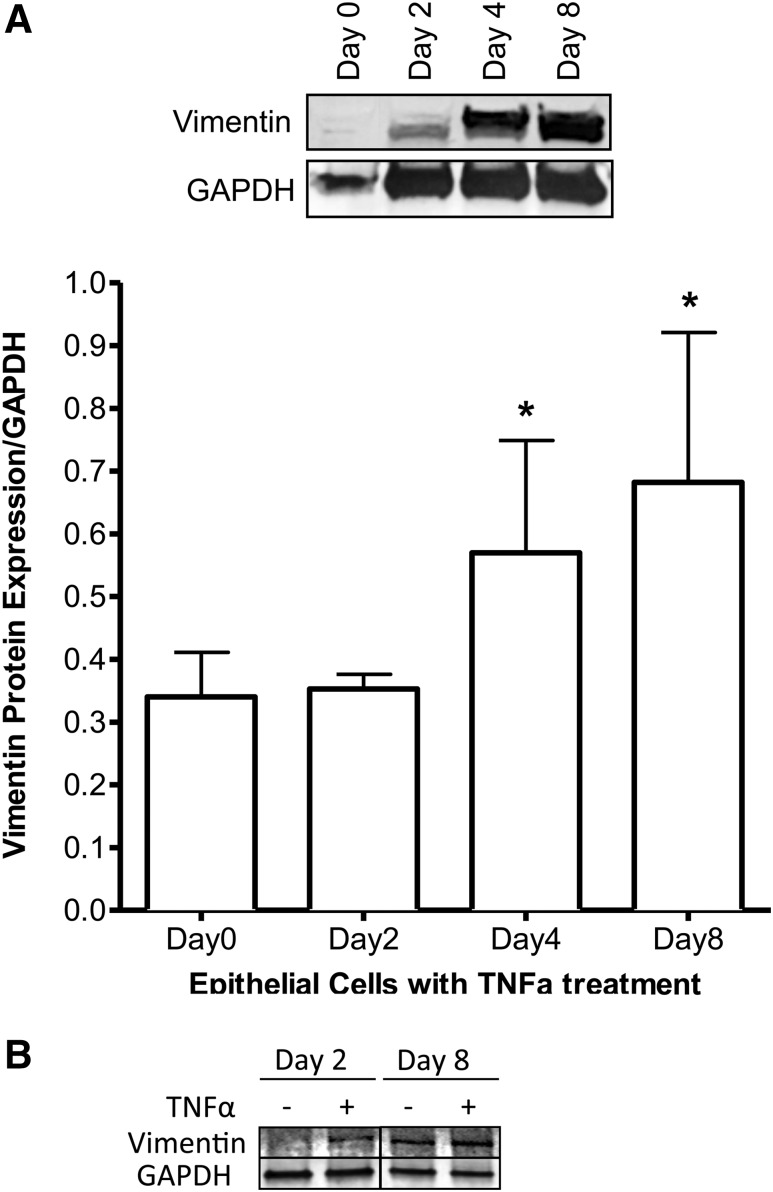
(A) Vimentin expression of epithelial cells treated with TNF-*α* over a period of days (n = 3). Day 0 begins when the samples have been cultured until the epithelial cells are 80% confluent. Vimentin is a marker for mesenchymal cells. GAPDH was used as a loading control. The cells are from full-term human placental amnion. *P < 0.05. (B) Representative blot showing untreated cells vs TNF-*α* treatment on days 2 and 8.

The proinflammatory cytokine, TNF-*α*, is an inducer of EMT in several cancers (15, 16). Because TNF-*α* is reported to be present in detectable levels in the amniotic fluid from VD and in significantly increased levels in the amniotic fluid from premature rupture of membrane and PPROM (17), we questioned if this cytokine could contribute to the induction of EMT in the amnion as well. We found that cell culture of epithelial cells derived from CS (n = 3) exhibited increased expression of the mesenchymal marker vimentin over time, as evidenced by immunoblot analysis [Fig. 3(A)]. Figure 3(B) is a representative western blot showing untreated/control cells on days 2 and 8. Vimentin expression was increased by TNF-*α* treatment at day 2 compared with control, but by day 8, even untreated cells in culture increased vimentin expression, similar to those treated with TNF-*α* (18).

### TNF-*α*–induced EMT promotes pressure-induced rupture of amniotic membranes

**Figure 4.**
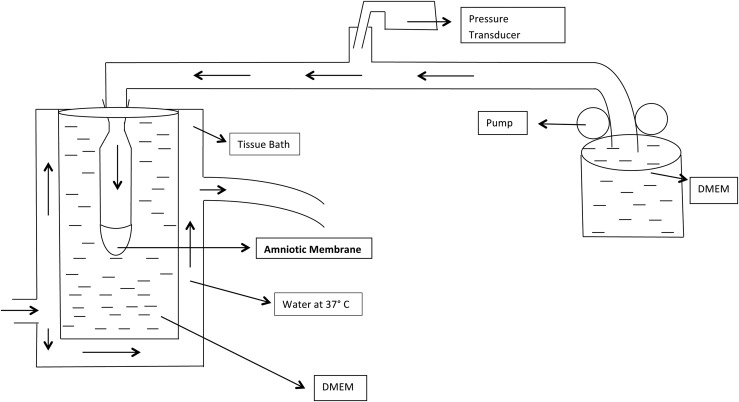
Schematic diagram of *in vitro* chamber with amniotic membrane under applied flow. The amniotic membrane is placed over a pressurized tube that pumps DMEM. The end of that tube is immersed in a DMEM bath at 37°C.

We next correlated TNF-*α*–induced EMT with pressure-induced rupture of membranes. We used an *in vitro* setup to study rupture of isolated amnion portions induced by the application of mechanical pressure. Amnion portions taken from CS (to ensure that they did not undergo prior EMT) were fixed tightly across an open-ended plastic tube, attached to a piston regulated by a syringe pump on the other end, creating a closed chamber, as depicted in Fig. 4. A dye was placed in the buffer enclosed in the chamber to monitor rupture on the application of pressure via the syringe pump. We observed that amnion exposed to TNF-*α* (known to induce EMT) ruptured at substantially decreased pressures compared with untreated amnion. Exposure to yet another pharmacologic EMT inducer, ethacridine, elicited a similar response.

### EMT inhibition protected amnion from rupture as measured by pressure and time

**Figure 5.**
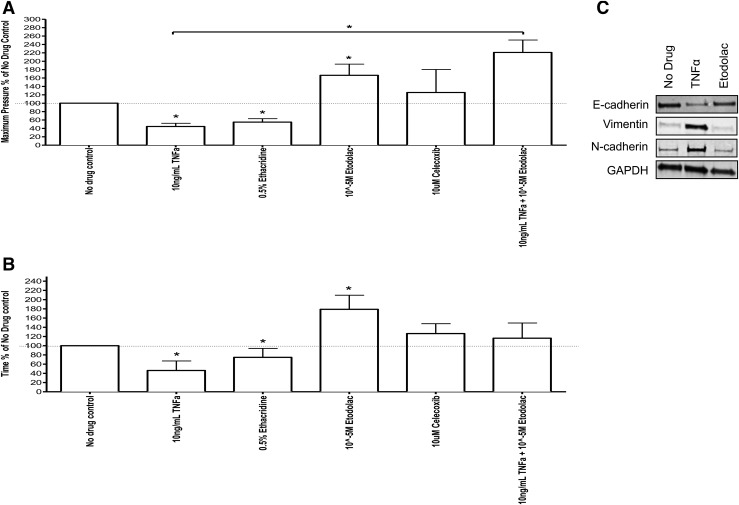
(A) Pressure required to rupture full-term placental amnion. Amnions from 3 CS-term pregnancies were incubated in media (no drug control), 10 ng/mL TNF-*α*, 0.5% ethacridine, 10^−5^ M etodolac, 10 μM celecoxib, and 10 ng/mL TNF-*α* with 10^−5^ M etodolac at 37°C overnight. Pressure to rupture was measured after treatment. *P < 0.05. (B) Time required to rupture full-term placental amnion. Amnions from 3 CS-term pregnancies were incubated in media (no drug control), 10 ng/mL TNF-*α*, 0.5% ethacridine, 10^−5^ M etodolac, 10 μM celecoxib, and 10 ng/mL TNF-*α* with 10^−5^ M etodolac at 37°C overnight. Time to rupture was measured after treatment. *P < 0.05. (C) Protein expression [after TNF-*α* (10 ng/mL) vs etodolac (10^−5^ M) treatment and rupture of amnion from 3 CSs] of E-cadherin, vimentin, and *N*-cadherin was measured in western blot. GAPDH was used as a loading control.

In contrast with exposure to TNF-*α*, amnion exposed to a *bona fide* EMT inhibitor, etodolac, required substantially increased pressure to rupture when compared with untreated controls. Because etodolac is also a known inhibitor of COX-2, we determined whether the inhibitory effect of etodolac (on pressure-induced amnion rupture) was independent of its inhibitory action on COX-2. To do this, we exposed amnion to another *bona fide* COX-2 inhibitor, celecoxib, which does not induce EMT (19). Under these conditions, we did not observe any substantial inhibition of pressure-induced rupture when compared with untreated controls. Moreover, pretreatment with etodolac followed by exposure to TNF-*α* reversed the effects of TNF-*α* alone, on the required pressure, to rupture membranes [Fig. 5(A)].

We also observed that membranes exposed to TNF-*α* ruptured at significantly lesser times compared with untreated controls when subjected to the same pressure. As expected, membranes exposed to etodolac took a significantly longer time to rupture compared with untreated controls when subjected to the same pressure. Pretreatment with etodolac reversed the effects of TNF-*α* alone [Fig. 5(B)].

Treatments with both TNF-*α* and ethacridine were associated with increased expression of the mesenchymal markers vimentin, *N*-cadherin, and fibronectin in the membrane portions (subjected to the aforementioned pressure-induced ruptures), further confirming that rupture of membranes at significantly lower pressures (compared with untreated controls) correlated with an increased mesenchymal phenotype [Fig. 5(C)].

### Amnion derived from PPROM exhibited increased EMT

**Figure 6.**
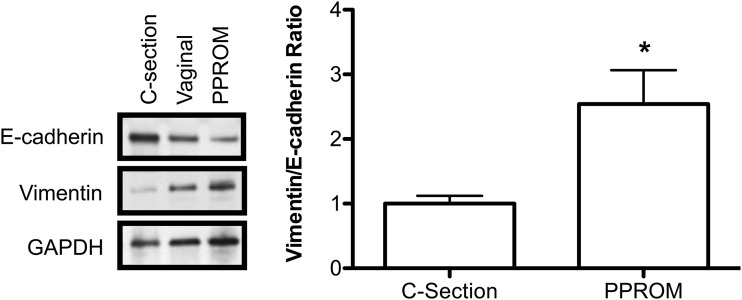
Total protein from amnions of CS-term pregnancy (n = 3), VD from term pregnancy (n = 3), and PPROM (n = 3). The gestational ages of the PPROM samples were 36 weeks, 34 weeks 5 days, and 36 weeks 3 days. Protein expression of E-cadherin and vimentin was measured in western blot. GAPDH was used as loading control. *P < 0.05.

Our data so far indicated that EMT is a fundamental biochemical event associated with pressure-induced rupture of amniotic membranes. Hence, we next assessed whether amnions obtained from PPROM cases exhibited increased EMT when compared with VD and CS. In this regard, we observed amnions from PPROM cases exhibited significantly increased expression of the mesenchymal marker vimentin compared with CS and normal VD. In contrast, they exhibited significantly decreased expression of the epithelial marker E-cadherin when compared with those from VD and CS (Fig. 6).

## Discussion

The primary objective of this work was to assess the role of EMT in the mechanism of rupture of the amniotic membrane. In this study, we observed that ruptured amnion obtained from normal VD at term exhibited significantly increased mesenchymal markers compared with those delivered by CS. This was the first indication that EMT could be associated with amniotic membrane rupture. Moreover, a significantly increased population of amnion-derived cells from term VDs expressed markers for both epithelial and mesenchymal characteristics when compared with CSs. This is most likely indicative of cells in the transition phase between pure epithelial and pure mesenchymal cell populations. Immunoblot analysis of mesenchymal and epithelial cell markers in intact membranes also exhibited a similar trend.

TNF-*α* has been shown to be a major inducer of EMT in cancer (6, 20). Interestingly, in 74 normal amniotic fluid samples taken for *α*-fetoprotein screening during the second and third trimesters, 67 (91%) contained TNF-*α*, with a mean concentration of 1.7 ng/mL (21). Moreover, relatively increased levels of TNF-*α* were also observed in amniotic fluids obtained from premature rupture of membrane and PPROM cases (17, 21, 22). However, whether TNF-*α* is at least in part responsible for EMT-associated rupture of amniotic membranes is not yet known. We therefore assessed whether TNF-*α* could induce EMT in isolated pure amniotic epithelial cells. Indeed, we observed that TNF-*α* induced increased expression of vimentin in amniotic epithelial cells, similar to its effect on epithelial cells derived from tumors (6, 20).

Traditionally, rupture of the fetal membranes has at least in part been attributed to increasing physical stresses during uterine contractions that weaken the membranes (23, 24). Several lines of evidence also indicate that mechanical pressure and the stretching of membranes are also associated with key biochemical events of amniotic membrane rupture at the molecular level (25, 26). We therefore assessed whether TNF-*α–*induced EMT was one of the critical molecular events which facilitated pressure-induced rupture of the amniotic membrane. We used an *in vitro* system which allowed us to study the effects of applied mechanical pressure on the rupture of membranes treated with or without various known inducers and inhibitors of EMT. Our observation that pretreatment of the amniotic membrane with TNF-*α* increased the sensitivity of membranes to pressure-induced rupture of amnions is consistent with our hypothesis that TNF-*α–*induced EMT plays a role in promoting pressure-induced amniotic membrane rupture.

We next assessed whether the TNF-*α*–facilitated pressure induced rupture of the amniotic membrane was at least in part caused by EMT. Our observation that celecoxib, a specific COX-2 inhibitor, did not attenuate the TNF-*α*–facilitated pressure-induced rupture of the amniotic membrane indicated that TNF-*α*–mediated inflammatory processes *per se* did not play a role in the rupture of the amniotic membrane. By contrast, etodolac, which is also a COX-2 inhibitor and a well-known inhibitor of EMT, significantly inhibited the TNF-*α*–facilitated pressure-induced rupture of the amniotic membrane. The COX inhibitors were used at concentrations that were significantly more than their reported IC50s (27–29) in inhibiting COX. The role of EMT in this process was further confirmed when ethacridine, a known inducer of EMT, potentiated the pressure-induced rupture of the amniotic membrane (30). Interestingly, in some countries, ethacridine has been successfully used as a drug to induce second trimester abortions (31).

Preterm rupture of membrane and PPROM are associated with ~30% to 40% of preterm deliveries and occur in ~1% to 3% of all pregnancies (32). These are associated with substantial fetal morbidities (32). Currently, there are no effective treatments for these pathophysiologic conditions (33–35). Our findings demonstrated that amnions derived from PPROM exhibited increased EMT. It is also known that increased oxidative stress is associated with EMT in cancer epithelial cells (33–35).

In conclusion, our study has identified EMT as another mechanism by which amniotic membranes could undergo pressure-induced rupture. Hence, inhibitors of EMT could be used to treat conditions related to early rupture of membranes. Hence, inhibitors of EMT could prove to be effective in the prevention of fetal morbidities associated with diseases related to preterm and premature rupture of the amnion.
